# Preparation and Characterization of Reduced Graphene Oxide /TiO_2_ Blended Polyphenylene sulfone Antifouling Composite Membrane With Improved Photocatalytic Degradation Performance

**DOI:** 10.3389/fchem.2021.753741

**Published:** 2021-10-19

**Authors:** Fengna Dai, Shangying Zhang, Qi Wang, Haiquan Chen, Chunhai Chen, Guangtao Qian, Youhai Yu

**Affiliations:** Center for Advanced Low-Dimension Materials, State Key Laboratory for Modification of Chemical Fibers and Polymer Materials, College of Material Science and Engineering, Donghua University, Shanghai, China

**Keywords:** polyphenylenesulfone, reduced graphene oxide, titanium dioxide, photodegradation, self-cleaning

## Abstract

Nanosized titanium oxide (TiO_2_)-based photocatalysts have exhibited great potential for the degradation of organic contaminants, while their weak absorption of visible light limits the photocatalytic efficiency. Herein, a novel reduced graphene oxide/TiO_2_-polyphenylenesulfone (rGO/TiO_2_-PPSU) hybrid ultrafiltration membrane has been successfully prepared via a non-solvent induced phase-separation method, in which the synergistic coupling between the rGO and TiO_2_ could endowed the fabricated membranes with visible-light-driven efficient photocatalytically degradation of organic pollutants and outstanding photocatalytic and antifouling properties. Compared with the PPSU membranes prepared with Graphene oxide and TiO_2_, respectively, the rGO/TiO_2_-PPSU membrane demonstrated significant photodegradation towards phenazopyridine hydrochloride (PhP) solution under ultraviolet light (improved about 71 and 43%) and visible light (improved about 153 and 103%). The permeability and flux recovery rates of the membrane indicated that the high flux of the rGO/TiO_2_-PPSU membrane can be greatly restored after fouling, due to the improved self-cleaning properties under visible light static irradiation. With the properties of high performance of photocatalytic degradation and good self-cleaning ability, the rGO/TiO_2_-PPSU membrane would have great potential in water treatment.

## Introduction

The development of sewage and wastewater treatment is of utmost importance to address water crisis and obtain clean and safe drinking water (Brende, 2020). Ultrafiltration membrane technology has emerged as a cost-effective and sustainable application method in sewage and wastewater treatment in light of its excellent performance in separating particulates, organic pollutants and inorganic components in water. The ultrafiltration membranes are often based on organic polymers including polysulfone (PSF), poly(ether sulfone) (PES), polyphenylsulfone (PPSU), polyacrylonitrile (PAN) and polyvinylidene fluoride (PVDF) (Dharupaneedi et al., 2019). Among them, PPSU has attained great attention as an ideal membrane polymer in the field of membrane separation technology with its rigidity, good fluidity, good chemical corrosion resistance and high mechanical strength. However, due to its hydrophobicity, PPSU is prone to membrane fouling, which weakens the separation performance of the membrane, shortens the service life of the membrane and increases operation and maintenance cost of membrane (Golpour and Pakizeh, 2018; Dai et al., 2019; Kumar et al., 2021). In addition, the traditional membrane separation technology lacked the ability of removing all organic pollutants. Therefore, it is significant to explore a novel separation membrane system which can effectively address these issues.

Photocatalytic membrane reactor (PMR) based on the degradation of organic pollutants in water and wastewater through oxidation reactions was a prospective technology in water separation and purification driven by UV or visible light and facilitated the alleviation of membrane fouling without excessive waste of chemicals (Iglesias et al., 2016; Zhang et al., 2016; Kumari et al., 2020). Generally, the PMRs can be divided into two configurations: powder photocatalyst suspended in the reactor (SPMR) and photocatalyst fixed on the membrane (IPMR) (Espíndola et al., 2019). For SPMR, the recovery and post-treatment are principal challenges limiting its application. IPMR integrated both separation and photocatalytic properties, are easier to be recycled. The photocatalytic membranes (PMs) are the core to supported PMRs, in which the photocatalytic nanoparticles were attached to the membranes. The photocatalytic composite membranes can be prepared through different methods such as physical blending (Singh et al., 2020; Meng et al., 2021), self-assembly (Li et al., 2019), layer-by-layer assembly (Yan et al., 2019), chemical grafting or deposition (Ma et al., 2014; Pei et al., 2021). Among these, the physical blending is an effective and convenient manner because the membrane preparation process does not require additional operations during or after the phase transformation (Barzegar et al., 2020; Singh et al., 2020).

Numerous semiconductor photocatalysts have been reported, including TiO_2_, ZnO, CdS, etc (Shen et al., 2020; Tran et al., 2020; Zangeneh et al., 2020; Yang et al., 2021). Among them, TiO_2_ has garnered tremendous attention for its low-cost, nontoxicity, high catalytic activity, high chemical stability, acid and alkali resistance, photochemical corrosion resistance, non-pollution and harmless to people (Mohd Hir et al., 2018; Zangeneh et al., 2019). When exposed to ultraviolet (UV) irradiation, TiO_2_ can effectively decompose most organic compounds. This is because the band gap energy of TiO_2_ is lower than the energy of ultraviolet radiation, which can excite titanium dioxide to produce photoelectrons (e^−^) and holes (h^+^). The e^−^ and h^+^ respectively react with O_2_ and H_2_O to form free radicals such as •O_2_^−^ and •OH. The strong oxidizing radicals (•O_2_^−^ and •OH) and h^+^ could directly participate in the decomposition of organic pollutants (Georg and James, 1995). Furthermore, the superhydrophilicity of TiO_2_ will modulate surface morphology, improve permeability, and boost the self-cleaning and antifouling ability of membranes (Romay et al., 2020). However, UV light is an important factor to accelerate the aging of polymer membrane and shorted the service life of photocatalytic membranes. Hence, it is vital to develop PMs working in sunlight to realize water purification and reduce membrane aging during prolonged use.

Recently, it has been shown that the combination of graphene and its derivatives with TiO_2_ is an effective way to reduce the electron hole recombination rate and improve its photocatalytic activity under visible light (Zhou et al., 2020; Hu F. et al., 2021; Xu et al., 2021). Graphene oxide (GO), obtained by chemically modified from graphene, is a novel material composed of sp^2^ hybridized carbon atoms packing tightly into a monolayer two-dimensional honeycomb lattice structure. It has been widely applied in photocatalysis because of its large specific surface areas, high thermal conductivity, fast electron mobility and strong Young’s modulus (Hu Y. et al., 2021). In rGO/TiO_2_ nanocomposites, the network structure of rGO with large surface area and oxygen-containing functional groups are idea platforms for the tight-binding with TiO_2_ to avoid the agglomeration of TiO_2_ and enhance the photocatalytic effect of rGO/TiO_2_. Moreover, the rGO nanosheets can accept the photoinduced electron conduction of TiO_2_ and inhibit the electron hole recombination at the same time (Hunge et al., 2020; Chen et al., 2021).

The purpose of this study is to develop a novel kind of reusable photocatalytic rGO/TiO_2_-PPSU hybrid UF membrane by physical blending and the non-solvent induced phase-separation method. The rGO/TiO_2_ nanocomposites and the hybrid membranes were prepared and characterized. Using phenazopyridine hydrochloride (PhP) as a model containment, the photocatalytic degradation ability of the prepared membranes under both UV and visible irradiation was tested. The separation performance, as well as the antifouling and self-cleaning properties of the hybrid membranes were also evaluated.

## Materials and Methods

### Materials

Polyphenylene sulfone (PPSU) was obtained from Shandong Horan Special Plastic Co., Ltd. (Weihai, China) 1-methyl-2-pyrrolidone (NMP) were obtained from Shanghai Lingfeng Chemical Co., Ltd. (Shanghai, China) Polyvinylpyrrolidone K30 (PVP K30, Mw = 44,000–54,000), Flake graphite (3,500 mesh), Potassium persulfate (K_2_S_2_O_8_), sulfuric acid (H_2_SO_4_, 98%), Nitric acid (HNO_3_, 68%), Phosphorus pentoxide (P_2_O_5_), Potassium permanganate (KMnO_4_) and hydrogen peroxide were acquired from Sinopharm Chemical Reagent Co., Ltd. (Shanghai, China) Titanium dioxide (TiO_2_, P25) was purchased from Degussa AG Co., Ltd. (Frankfurt, Germany) Phenazopyridine hydrochloride (PhP) was obtained from Adams Reagent Co., Ltd. (Shanghai, China) Characteristics of phenazopyridine hydrochloride were shown in Table 1. All reagents were applied without further purification.

**TABLE 1:** Characteristics of phenazopyridine hydrochloride.

Structure	Formula	λ_max_ (nm)	M_w_ (g/mol)	Solubility in Water (g/L, 25°C)
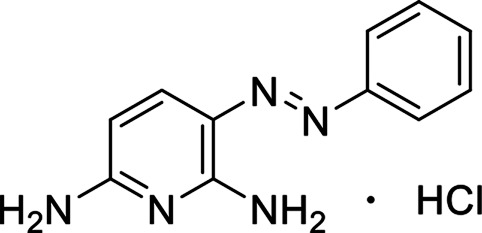	C_11_H_12_N_5_Cl	428	249.7	15.9

### Synthesis of GO and rGO/TiO_2_ Nanocomposites

GO was synthesized first by natural graphite powder via a modified Hummer’s method (Hummers and Offeman, 1958; Ding et al., 2019). In brief, 5 g graphite was uniformly dispersed in 300 ml of concentrated H_2_SO_4_ in a round-bottom flask, stirring for 5 min. After that, 4.2 g K_2_S_2_O_8_ and 6.2 g P_2_O_5_ were slowly added, the mixture was heated and stirred at 80°C for 5 min. After filtration and drying, the product was transferred to a 200 ml beaker of concentrated H_2_SO_4_, and 15 g KMnO_4_ was slowly added and vigorous agitation in an ice bath. The resulting mixture was treated with H_2_O_2_ until the gas ceased to be emitted. The resulting suspension was thoroughly washed, first three times with a diluted hydrochloric acid solution, then filtered with deionized water and centrifuged until the supernatant reached a pH of 7. The suspension was lyophilized to obtain pure GO powder.

The rGO/TiO_2_ composites was prepared via a facile one-step hydrothermal method (Zhang et al., 2010). Basically, 22.5 mg GO was dispersed into a mixture of ethanol/water (2:1 v/v) and ultrasonicated for 60 min. After dispersing, 202.5 mg TiO_2_ powder was added to agitate intensely for 5 h for a complete homogenization. Finally, a 30 ml mixture was transferred into a 100 ml Teflon-lined stainless autoclave and attained 120°C for 5 h. The obtained product was recovered through centrifuged with deionized water and ethanol and freeze-dried.

### Preparation of rGO/TiO_2_ Hybrid Ultrafiltration Membranes

The preparation of hybrid PPSU ultrafiltration membranes by solution casting and the non-solvent induced phase-separation method were based on previous study with a slight modification (Wang et al., 2021). Briefly, the casting solution, consisting of PPSU, the rGO/TiO_2_ nanocomposites, PVP K30 (used as the porogen) and NMP (used as the solvent), were prepared under ultrasonication to obtain a homogeneous mixture, and then the mixture was further mechanical stirred at 300 rpm for 12 h. After bubble removal for 30 min, the casting solution was poured onto a clean glass plate with a scraper of 150 μm and immediately transferred to a water coagulation bath at 25°C until the membrane peeled off. Before testing, the resulting membranes were maintained in deionized water and replaced the water every 12 h. For comparison, GO-PPSU membranes, TiO_2_-PPSU membranes and pristine PPSU membranes were prepared with the same procedure as mentioned above. The recipes of the membranes are shown in Table 2.

**TABLE 2:** The recipes of the membrane casting solutions.

Membranes	Mass ratio (wt%)			
	Nanomaterials	PPSU	PVP K30	NMP
PPSU	—	20	1	79
GO-PPSU	0.8	20	1	78.2
TiO_2_-PPSU	0.8	20	1	78.2
rGO/TiO_2_-PPSU	0.8	20	1	78.2

### Characterization of GO and rGO/TiO_2_ Nanocomposites

The morphology and microstructure of the GO and rGO/TiO_2_ nanocomposites were characterized on the scanning electron microscopy (SEM; Regulus8230), transmission electron microscopy (TEM; JEM-2100) and high-resolution TEM (HRTEM; TecnaiG2F20). The crystalline structure of samples was observed by X-ray diffraction (XRD; RIGAKU, D/max-2550VB + /PC) equipped with Cu Ka radiation (18 kW, 10° ≤ 2θ ≤ 90°). The chemical composition of the membranes was characterized by using X-ray photoelectron spectroscopy (XPS, Thermo Scientific, Escalab 250Xi) provided with Al Kα source and Fourier transform infrared spectroscopy (FTIR; Bruker, VERTEX70) using KBr tabletting method. The phase structure was observed by laser Raman spectrometer (Renishaw inVia-Reflex) using a 532 nm laser line in the wavelength range of 100–3,000 cm^−1^ at room temperature.

### Characterization of Membranes

#### Membrane Characterization Techniques

The optical properties of hybrid membranes were investigated by using ultraviolet-visible diffuse reflection spectroscopy (UV-Vis DRS; PerkinElmer Instrument, 1901 Lambda 950). The top surface and cross section morphology of hybrid membranes were investigated by SEM after fracturing in liquid nitrogen and gold spraying. Energy dispersive X-ray (EDX) spectroscopy attached to SEM was employed to analyze the element composition. The surface hydrophilicity of hybrid membranes was detected by the water contact angle (WCA) using Drop Shape Analysis Data physics (OCA 20) at room temperature.

#### Permeation and Rejection of Membranes

Before measuring the pure water flux and PHP rejection, fabricated membranes were preloaded with DI water under the pressure of 0.2 MPa for 30 min. The permeate volume through the membrane was surveyed at 0.1 MPa, in which the feed solution was DI water and the PhP with a concentration of 15 mg/L respectively. The pure water flux (*J*_*0*_) and PhP rejection (*R*) were calculated according to the following Eqs 1–2:

$$J_{w}=Q/\left(A\times \Delta T\right),$$ (1)

where Q is the volume of the penetrating fluid (L), A the effective area of the membrane (m^2^), and ∆T the filtration time (h).

$$R\left(\%\right)=\left(1-C_{p}/C_{f}\right)\times 100\%,$$ (2)

where *C*_*p*_ (mg/L) and *C*_*f*_ (mg/L) are the PhP concentrations in the permeate and feed solution, respectively.

#### Photocatalytic Performance of Membranes

The photocatalytic performance of rGO/TiO_2_-PPSU membranes and the three control membranes were measured by decomposing PhP under the condition of dark, UV light and visible light. The membranes were immersed into the reactor containing 150 mL of 15 mg/L PhP solution. Before photocatalytic experiment, the solution containing membranes was keep in darkness for 60 min to access adsorption equilibrium, and then the system was exposed to UV irradiation (250 W mercury lamp) and visible irradiation (300 W Xenon lamp) for photocatalytic degradation. The concentrations of PhP were measured every 30 min using an ultraviolet spectrophotometer (PerkinElmer Precisely, Lambda950) at 428 nm. The PhP oxidation/mineralization tests were conducted by total organic carbon analyzer (TOC, Multi N/C 3100). The mineralization efficiency of PhP can be measured using Eq. 3:

$$TOC\;removal\left(\%\right)=\left[\;\left(TOC_{0}/TOC_{t}\right)/\;TOC_{0}\right]\times 100\%.$$ (3)

### Anti-fouling Performance of Membranes

The flux recovery rate (FRR) is an important parameter employed to evaluate the antifouling performance of membrane. The pure water flux (*J*_*0*_) of membranes were measured by the above steps. Then, the 15 mg/L PhP was served as feed solution to receive the flux of PhP solution (*J*_*1*_) after 120 min. Afterwards, the fouled membrane surface was rinsed with DI water to remove loose pollutants, and the water flux of the rinsed membranes (*J*_*2*_) was measured. Finally, the membranes were irradiated respectively under UV light and visible light for 30 min to remove contaminants on the membrane surface, and then test the water flux of the final clean membrane (*J*_*3*_). The total fouling ratio (R_t_), reversible fouling (R_r_), irreversible fouling (R_ir_) and FRR was calculated using Eqs 4–7:

$$\;FRR\left(\%\right)=\left(J_{3}/J_{0}\right)\times 100\%,$$ (4)

$$\;R_{r}\left(\%\right)=\left[\left(J_{3}-J_{1}\;\right)\;/J_{0}\right]\times 100\%,$$ (5)

$$R_{ir}\left(\%\right)=\left[\left(J_{0}-J_{3}\right)/J_{0}\right]\times 100\%,$$ (6)

$$R_{t}=R_{r}+R_{ir}.$$ (7)

## Results

### Characterizations

#### Morphology

**FIGURE 1:**
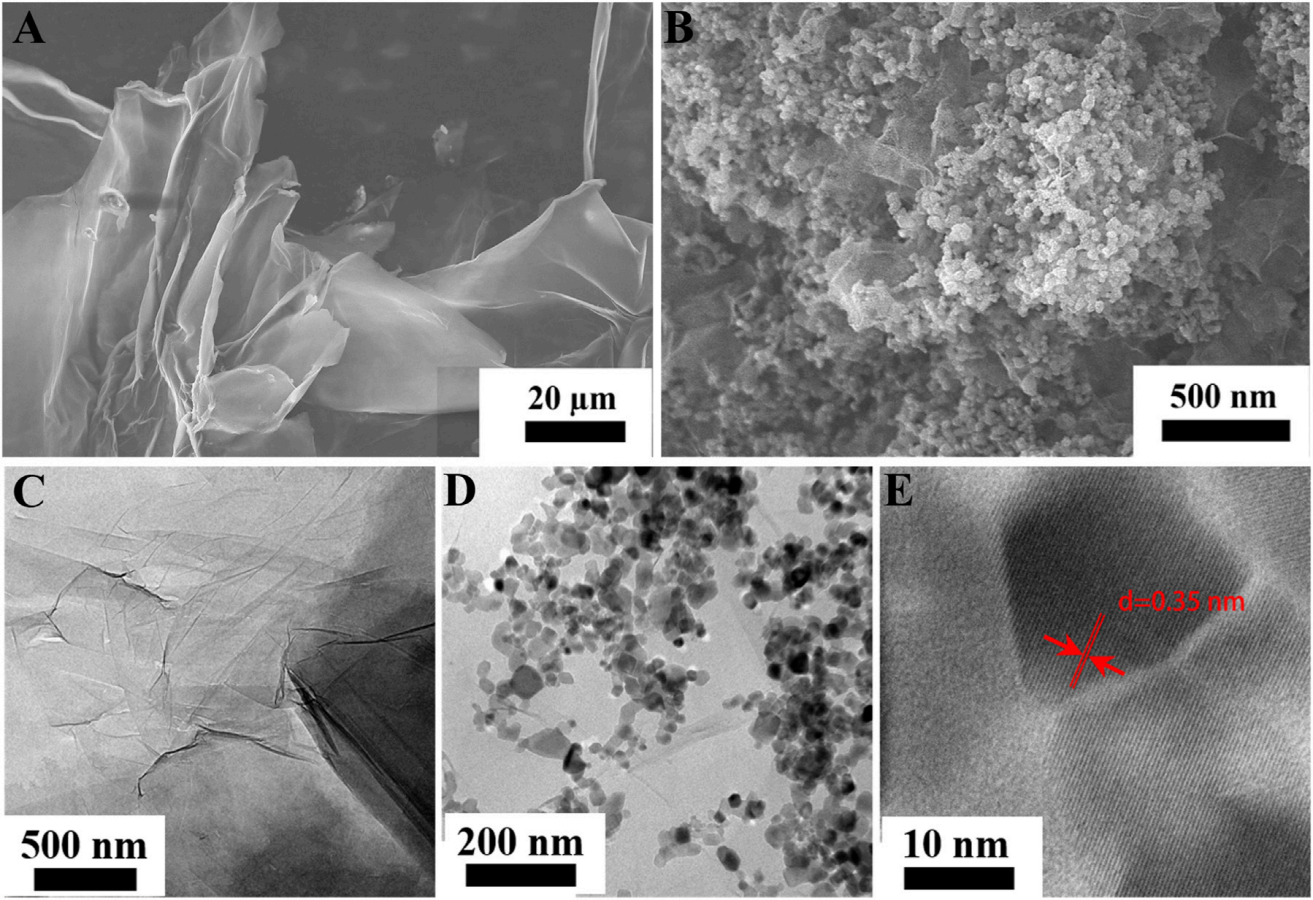
SEM **(A)** and TEM **(C)** images of GO; SEM **(B)**, TEM **(D)** and HRTEM **(E)** images of rGO/TiO_2_ nanocomposites.

The microstructure of GO and rGO/TiO_2_ nanocomposites was well examined by SEM and TEM are shown in Figure 1. In Figures 1A,C, the wrinkle and wormlike structures on GO surface indicate the successful preparation of GO. The SEM and TEM images of rGO/TiO_2_ nanocomposites exhibited that TiO_2_ were uniformly dispersed on GO in nanoscale dimension (Figures 1B,D). Dispersed TiO_2_ nanoparticles on the surface of the GO sheets may prevent the stack of the GO sheets, while recombination between the GO sheets and the TiO_2_ nanoparticles may weaken the aggregation of the TiO_2_ nanoparticles (Liu et al., 2021). The corresponding HRTEM images (Figure 1E) revealed that the (101) lattice spacing of TiO_2_ nanoparticles was estimated about 0.35 nm, which indicates that the TiO_2_ nanoparticles on GO sheets have fairly good crystallinity. The above SEM and TEM analysis showed that the TiO_2_ nanoparticles were firmly attached on GO sheets, which was beneficial to the photodegradation activity of rGO/TiO_2_ nanocomposites.

**FIGURE 2:**
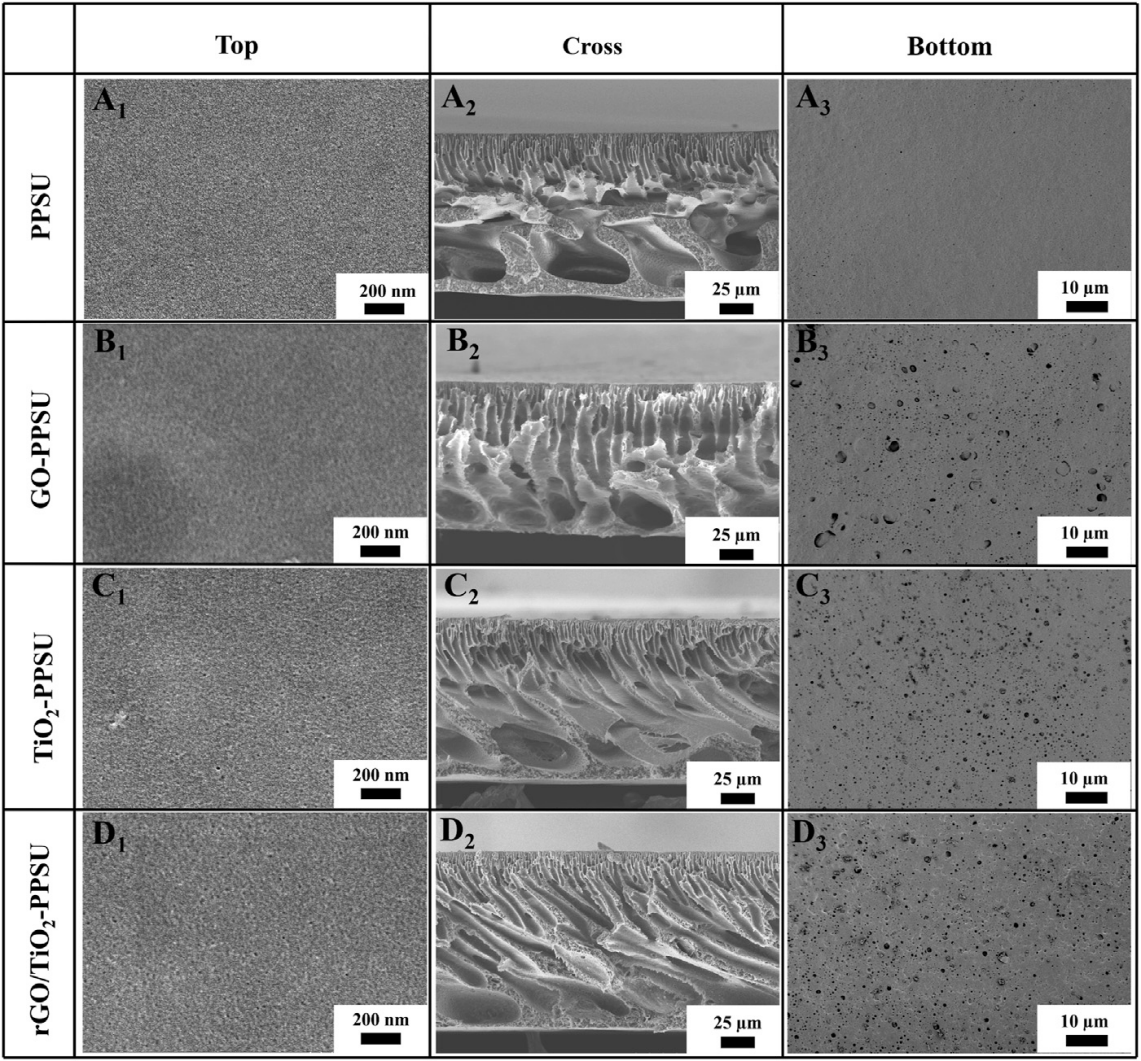
SEM images of top, cross-sectional and bottom surface morphology: pristine PPSU (A_1_, A_2_, A_3_), GO-PPSU (B_1_, B_2_, B_3_), TiO_2_-PPSU (C_1_, C_2_, C_3_) and rGO/TiO_2_-PPSU (D_1_, D_2_, D_3_) membranes.

**FIGURE 3:**
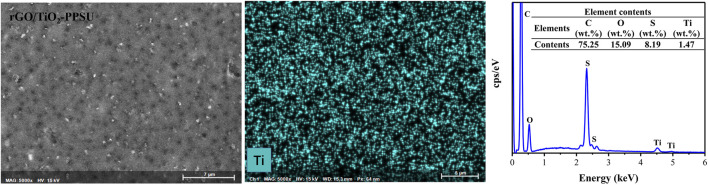
SEM-EDX images of rGO/TiO_2_-PPSU hybrid membranes.

Figure 2 illustrated the top, bottom and cross section SEM views of pristine PPSU, GO-PPSU, TiO_2_-PPSU, and rGO/TiO_2_-PPSU membranes. The cross-section SEM images presented that the typical asymmetric porous structure consisting of the thin and dense skin layers, finger-like porous supporting layers, macroporous and sponge-like structure intermediate layers existed in all the fabricated membranes. With the supplementation of TiO_2_, GO and the rGO/TiO_2_ nanocomposites into the system, the finger-like porous of the hybrid membranes became wider, whereas larger-size pore and multiple pore numbers appeared on the top and bottom surface compared with the pristine PPSU membrane, which should be the result of faster exchange rate between solvents and non-solvents caused by increased thermodynamic instability. These meliorated typical structures played a critical role in enhancing the transmembrane transport capacity of water and improving the antifouling performance of membranes. The formation of the larger-sized pores was brought about for the reason that the affinity between water and TiO_2_, GO and the rGO/TiO_2_ nanocomposite was higher than that between PPSU and water, which caused the permeation rate and exchange rate of solvent and non-solvent in the process of liquid-liquid phase transformation can be accelerated by mixing hydrophilic TiO_2_, GO and the rGO/TiO_2_ with PPSU, respectively (Xu et al., 2017). The SEM-EDX data of rGO/TiO_2_-PPSU membranes reflected the ingredients of Ti elements (Figure 3), which indicated the existence of rGO/TiO_2_ nanocomposites in the hybrid membranes.

#### Crystallinity

**FIGURE 4:**
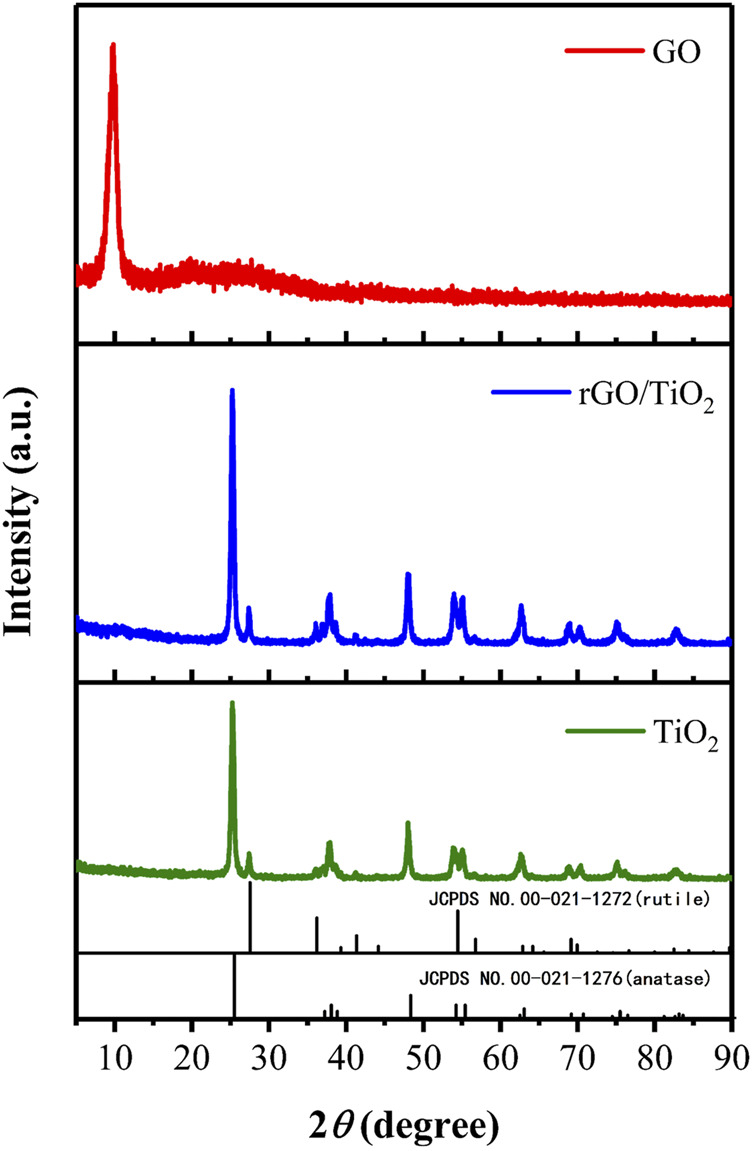
XRD patterns of GO, TiO_2_ and rGO/TiO_2_ nanocomposites.

Figure 4 indicates the XRD patterns of GO, TiO_2_ and rGO/TiO_2_ nanocomposites. A sharp peak (2θ ≈ 9.84°) in the XRD pattern of GO sheets corresponded to the (001) inter-layer structure of GO sheets. The TiO_2_ applied in this study consisted of a mixture of anatase type and rutile type, thus we infer that some characteristic peaks belonging to rutile TiO_2_ (JCPDS cards No. 00-021-1276) at 2θ of 27.41° (110) and several characteristic peaks of anatase TiO_2_ (JCPDS cards No. 00-021-1272) at 2θ of 25.29° (101), 37.87° (004), 48.14° (200), 54.01° (105), 55.05° (211), and 62.79° (204), 68.08° (116), 70.54° (220), 75.17° (215), 82.71° (224) in the XRD pattern of TiO_2_ (Zhang et al., 2021). There are no obvious differences between the XRD pattern of TiO_2_ and rGO/TiO_2_, while the (001) diffraction peak of GO disappeared, which means that the insertion of TiO_2_ may has disorganized the neat arrangement of GO sheets.

#### Chemical Composition

**FIGURE 5:**
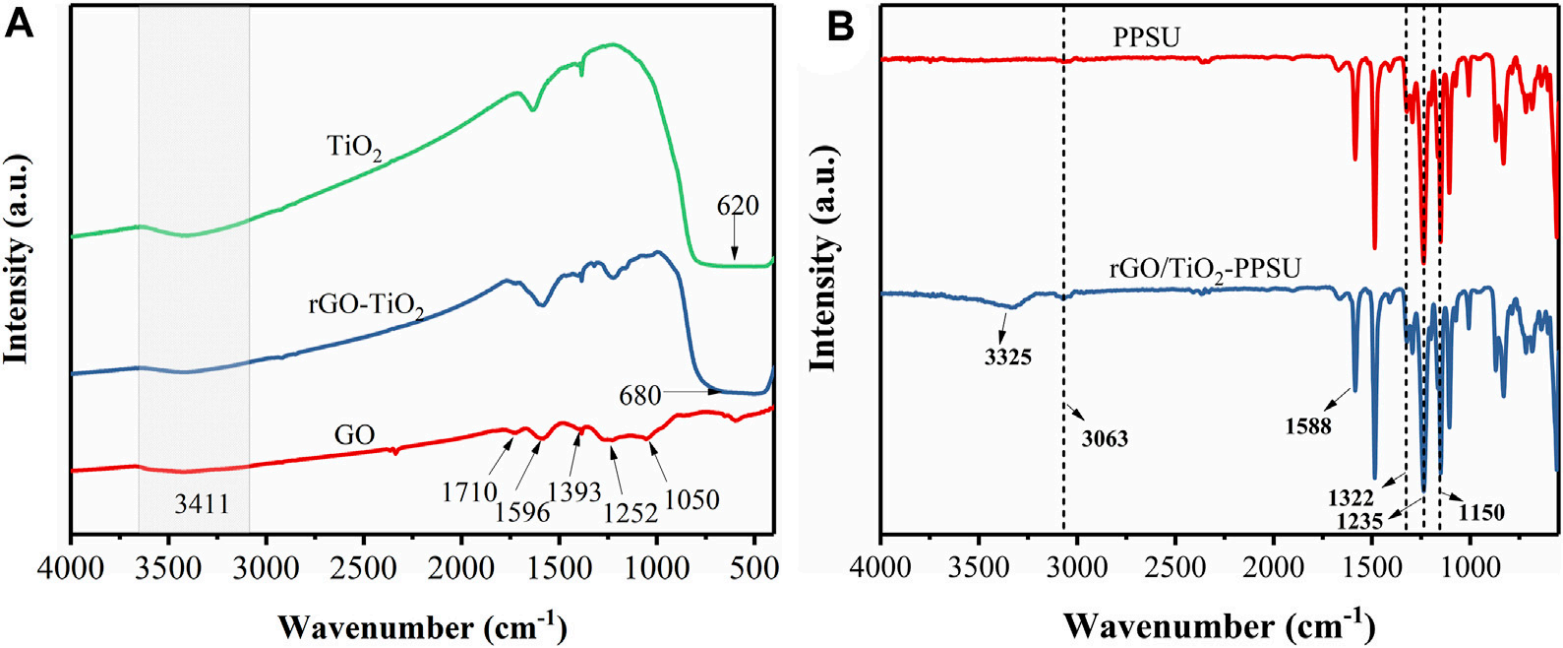
FTIR spectra of **(A)** GO, TiO_2_ and rGO/TiO_2_ nanocomposites; **(B)** pristine PPSU and rGO/TiO_2_-PPSU membranes.

The FTIR spectrum of GO, TiO_2_ and rGO/TiO_2_ nanocomposites revealed in Figure 5A. For GO, the absorption peaks at 1,050, 1,252 and 1,393 cm^−1^ owing to stretching mode of hydroxyl groups, epoxy C-O and O-H deformation, respectively. The characteristic peaks of C=O at 1,596 and 1,717 cm^−1^ were assigned to the skeletal vibration of unoxidized graphitic domains and stretching carboxylic groups located on the edges of the graphene oxide sheets. The O-H stretching of GO appeared in the broad absorption band at 3,411 cm^−1^. In the TiO_2_ spectra, a wide absorption band about 620 cm^−1^ corresponded to Ti-O-Ti stretching modes and a broad peak at 3,411 cm^−1^ belong to the surface-adsorbed water and hydroxyl groups. With respect to rGO/TiO_2_ nanocomposites, it can be seen that the majority of the GO peaks in the FTIR spectrum. The principal reason was that the formation of rGO during the hydrothermal synthesis process resulted in the disappearance of partial oxygen-containing functional groups on GO surface. In addition, the strong absorption band from 450 to 1,000 cm^−1^ was remarkably expanded with the introduction of GO, confirming the presence of both the Ti-O-Ti and Ti-O-C bonds in the nanocomposites (Nguyen et al., 2020). As is shown in Figure 5B, the chemical structures of pristine PPSU and rGO/TiO_2_-PPSU membranes were further identified by FTIR. The same absorption peak at 1,150 and 1,322 cm^−1^ are corresponded to symmetric and asymmetric O=S=O stretches, and the peak at 1,235 cm^−1^ can be assigned to the stretching C-O-C vibration. The peak at 1,588 cm^−1^ can be attributed to the stretching C=O. The absorption peaks at 2,913 cm^−1^ and 3,058 cm^−1^ contributed to the aromatic and aliphatic stretching vibrations of -CH_2_. Moreover, with the supplementary of the rGO/TiO_2_ nanocomposites, a broad absorption peak at approximately 3,325 cm^−1^ due to the O-H stretching vibration in the rGO/TiO_2_nanocomposites emerged, which indicates the enhanced hydrophilicity of the hybrid membranes.

**FIGURE 6:**
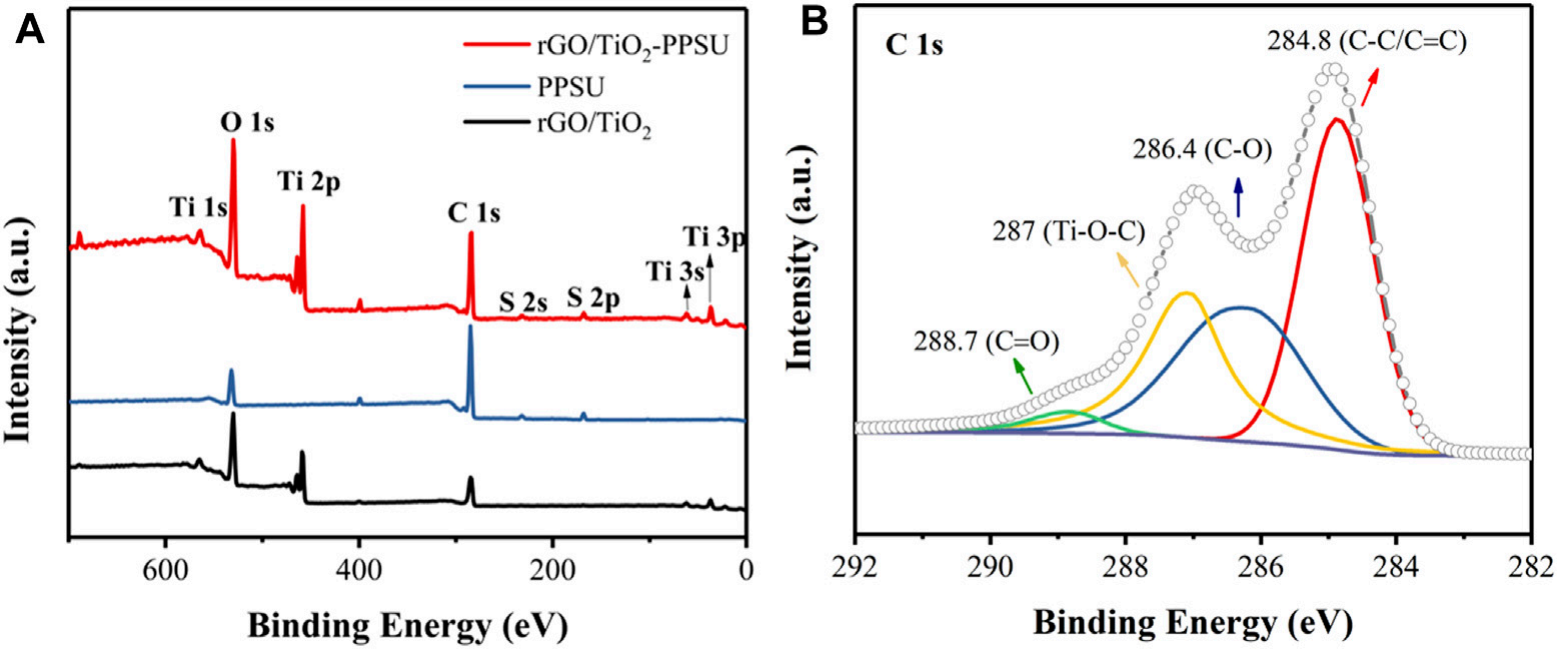
**(A)** XPS survey of rGO/TiO_2_ nanocomposites, PPSU and rGO/TiO_2_-PPSU membranes; **(B)** C 1s peaks of rGO/TiO_2_ nanocomposites.

XPS spectra of rGO/TiO_2_ nanocomposites, PPSU and rGO/TiO_2_-PPSU membranes was showed in Figure 6. The peak at 458 eV in the XPS full survey (Figure 6A) and the peak at 287 eV in C 1s spectrum (Figure 6B) were major evidence for the existence of Ti and Ti-O-C bond, respectively. These results support the successful preparation of rGO/TiO_2_ nanocomposites by hydrothermal synthesis. The XPS surveys in Figure 6A showed that the rGO/TiO_2_-PPSU composite membrane consisted of the elements (C, O, S, and Ti) of both PPSU and rGO/TiO_2_, which confirmed the successful immobilization of rGO/TiO_2_ onto PPSU membranes.

#### Optical Properties

**FIGURE 7:**
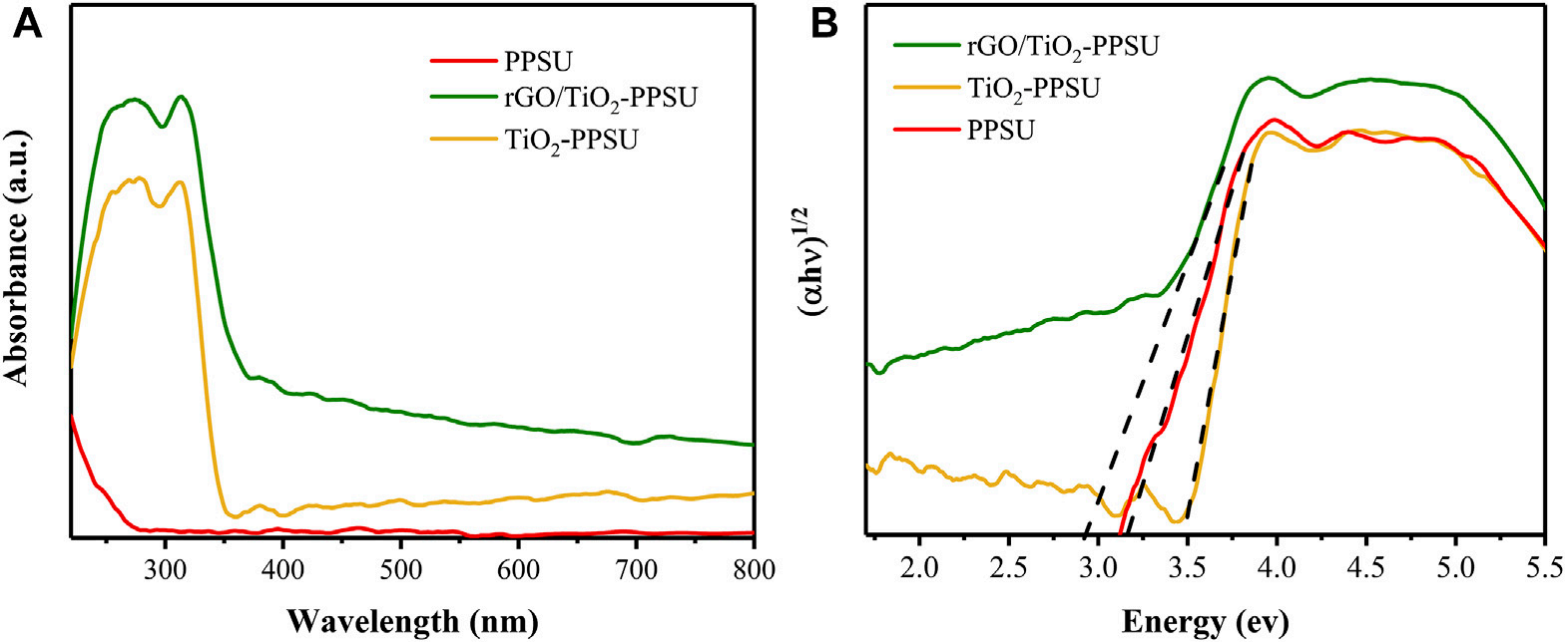
**(A)** UV-DRS spectrum of PPSU, TiO_2_-PPSU and rGO/TiO_2_-PPSU membranes and **(B)** Corresponding plot of transformed Kubelka-Munk function versus the light energy from **(A)**.

The UV–vis diffused reflectance spectra (UV-vis DRS) for the membrane of PPSU, TiO_2_-PPSU and rGO/TiO_2_-PPSU were shown in Figure 7A. The TiO_2_-PPSU membrane performed a typical absorption band ca. 360 nm, while the rGO/TiO_2_-PPSU membrane displayed a broad visible light absorption in the range of 400–800 nm because of the intercalation of GO, implying that the TiO_2_-PPSU membrane was photocatalytically active under visible light (Xu et al., 2016). The corresponding band-gap values of the TiO_2_-PPSU (3.48 eV) and rGO/TiO_2_-PPSU (2.8 eV) membranes were extrapolated from the Kubelka-Munk function based on the reflectance values (Figure 7B), which was conducive to a more efficient use of visible light. Moreover, the pristine PPSU membrane has a short adsorption wavelength and a large band gap in connection with its high dielectric strength (14.6 kV/mm) and volume resistivity (9 × 10^15^ Ω•cm). On the whole, the combination of rGO and TiO_2_ altered its crystalline and electronic structures.

### Photocatalytic Performance of Membranes

**FIGURE 8:**
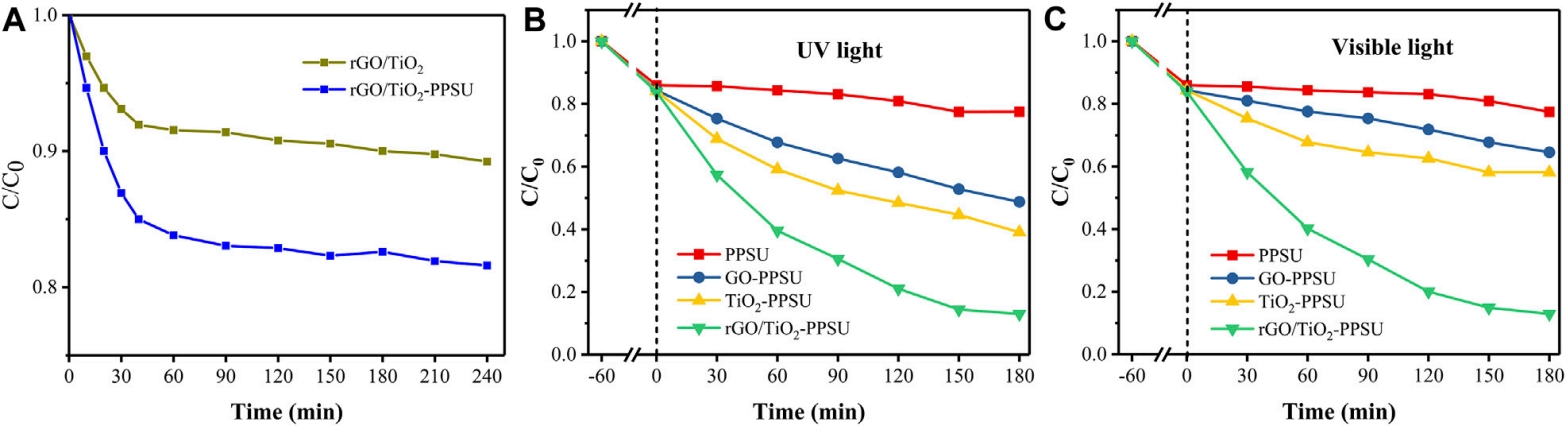
**(A)**The adsorption activity of PhP by the rGO/TiO_2_ and rGO/TiO_2_-PPSU hybrid membrane; Photodegradation of PhP under **(B)** UV light and **(C)** visible light for membranes.

The adsorption-desorption process of the membrane in PhP solution was studied before the photocatalytic performance experiment. Compared with the rGO/TiO_2_, rGO/TiO_2_-PPSU membrane showed much higher adsorption capacity (Figure 8A). The result can be ascribed to twofold: firstly, although PPSU membranes are highly hydrophobic, the supplementary of rGO/TiO_2_ caused a stronger adsorption of PhP solution on the membrane surface. Secondly, the unique porous structure of the membrane was also a vital parameter to promote adsorption. During the first 40 min of adsorption, the removal of PhP showed a rapid increase. After 40 min, the adsorption leveling off. Therefore, we selected 60 min of dark adsorption as the adsorption-desorption equilibrium point with the follow-up work.

**FIGURE 9:**
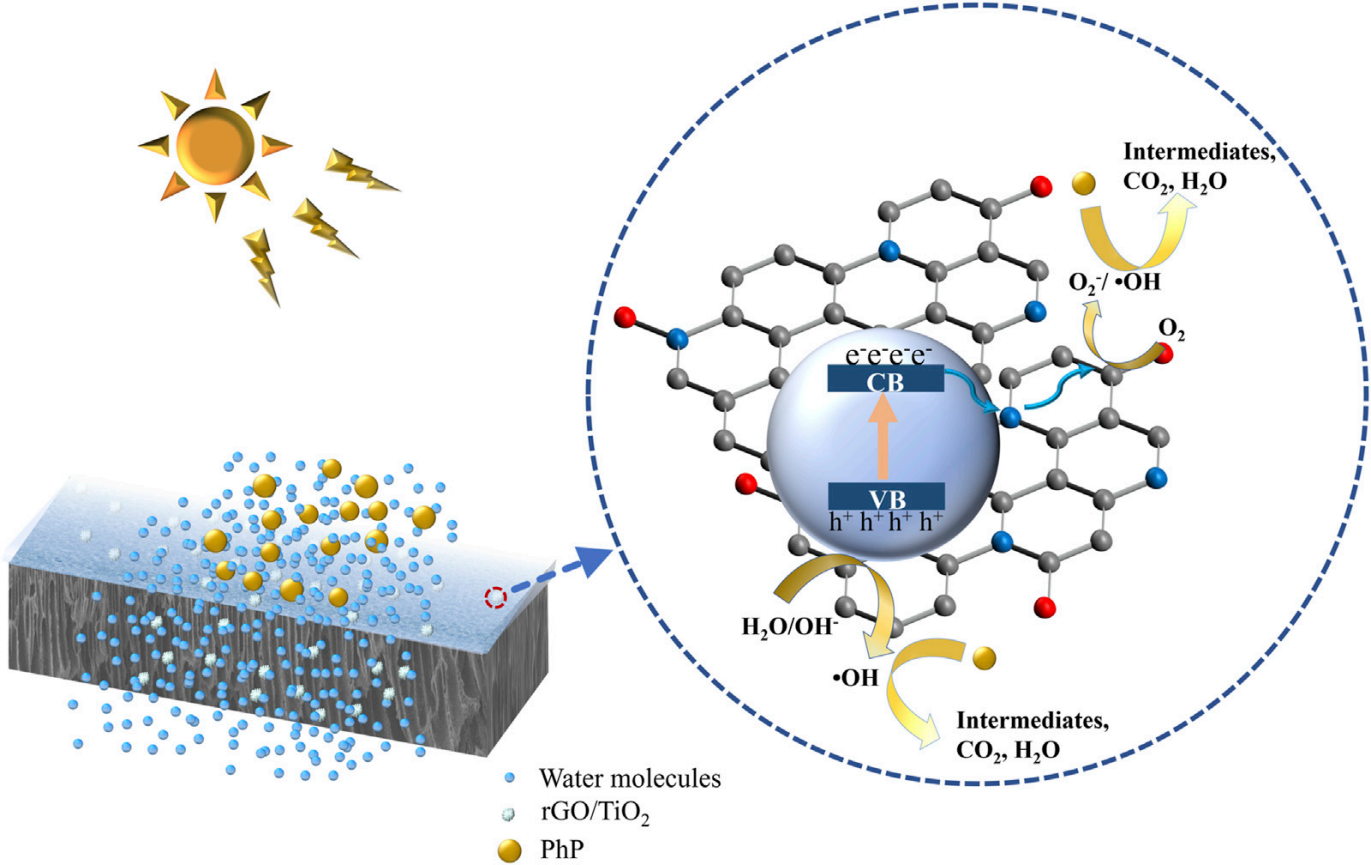
Schematic illustration for photocatalysis of rGO/TiO_2_-PPSU membrane.

The photocatalytic activity of rGO/TiO_2_-PPSU membrane was estimated by monitoring the decomposition of PhP under UV and visible irradiation. The reference samples of pristine PPSU, GO-PPSU and TiO_2_-PPSU membranes were studied under identical conditions, and the results were shown in Figure 8. As shown in Figures 8B,C, almost no photodegradation activity of PhP under UV and visible irradiation was observed for pristine PPSU membranes, suggesting that PPSU itself has practically no photocatalytic capability. By contrast, the hybrid membranes exhibited amplified photocatalytic activity doped with the TiO_2_, GO and the rGO/TiO_2_ nanomaterials under UV light. However, the GO-PPSU and TiO_2_-PPSU membranes showed only limited decomposition of PhP relative to the rGO/TiO_2_-PPSU membranes under visible irradiation. The main reason was that the wide band-gap energy of TiO_2_ limits its photocatalytic activation only by UV irradiation. Notably, GO-PPSU membranes showed a fairly similar photocatalytic performance to TiO_2_-PPSU membranes, suggesting that GO, as a semiconductor and photocatalyst itself, has a band-gap energy similar to TiO_2_, which has also been reported by previous study (Gao et al., 2014). The rGO/TiO_2_-PPSU membrane improved the removal rate of PhP, which was mainly related to the GO nanosheets supporting electron transfer and separation owing to their high charge mobility. The rGO/TiO_2_-PPSU membrane showed higher photocatalytic activity both under UV and visible light for 180 min, and the PhP removal efficiency reached 87 and 87.1%, respectively. In brief, the combination of TiO_2_ and rGO was beneficial to reducing the recombination effect of charge, narrowing the band-gap energy, expanding the light response range of the composite membranes to both UV and visible light range, and eventually improving the photocatalytic efficiency. The PhP photodegradation mechanism by the photocatalytic rGO/TiO_2_-PPSU hybrid membrane could be illustrated in detail by Figure 9 and Eqs 8–11. First, the hybrid membrane surface and pores were attracted with PhP molecules by the electrostatic adsorption. Then, the rGO/TiO_2_ nanoparticles on the hybrid membrane were excited to produce photoelectrons (e^−^) and holes (h^+^) under UV/visible irradiation, which migrated to the surface of rGO/TiO_2_. Finally, the e^−^ and h^+^ respectively react with •O_2_^−^ and H_2_O to form free radicals such as •O_2_^−^ and •OH. The strong oxidizing radicals (•O_2_^−^ and •OH) and h^+^ could directly oxidize PhP into the intermediates, CO_2_ and H_2_O.

$$\text{rGO}/{\text{TiO}}_{2}-\text{PPSU}+h\upsilon \to e^{-}+h^{+},$$ (8)

$${\text{e}}^{-}{+\text{O}}_{2}\to •{\text{O}}_{2^{-}}/•\text{OH},$$ (9)

$${\text{h}}^{+}{+\text{H}}_{2}\text{O}\to •{\text{OH}+\text{H}}^{+},$$ (10)

$$•{\text{O}}_{2^{-}}/•\mathrm{OH}/h^{+}+\;\text{PhP}\to \text{intermediates,}\;{\text{CO}}_{2}\;\text{and}\;{\text{H}}_{2}\text{O}\text{.}$$ (11)

### Permeability and Selectivity of Membranes

**FIGURE 10:**
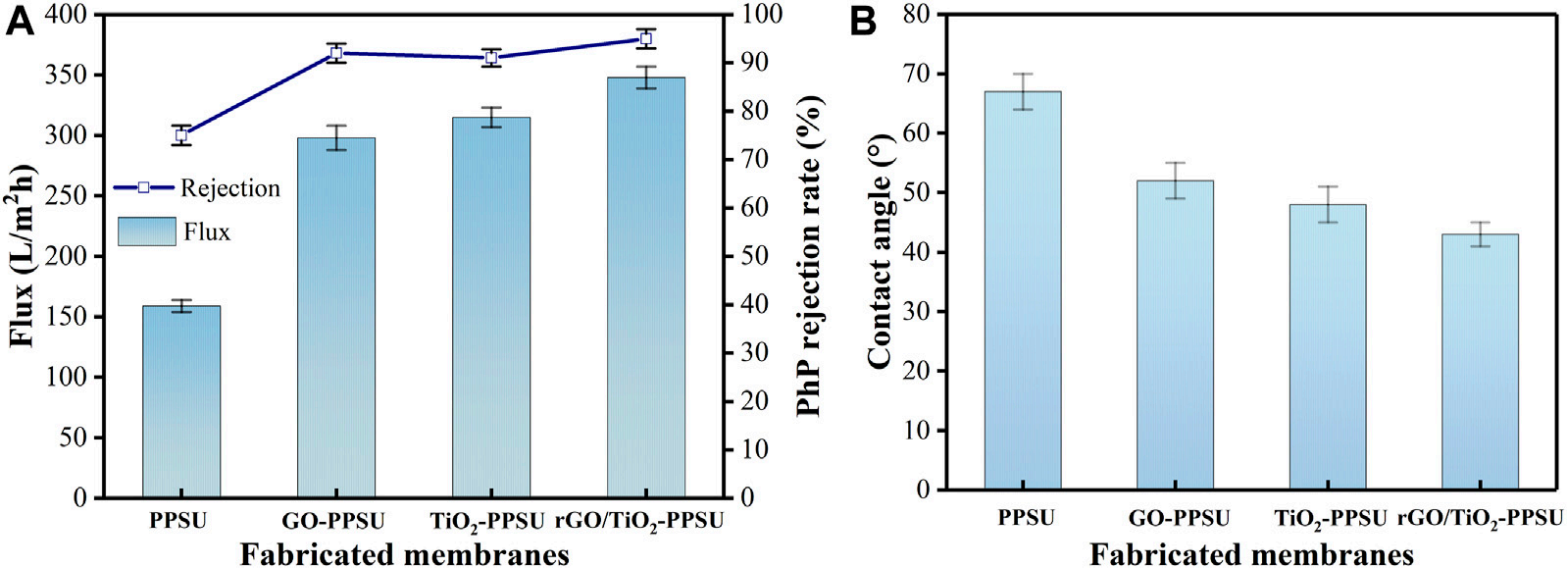
**(A)** The pure water flux and PhP rejection and **(B)** contact angle of membranes.

The permeability and selectivity of the rGO/TiO_2_-PPSU membrane were measured by judging the pure water flux and PhP rejection. As shown in Figure 10A, all the hybrid membranes doped with inorganic nanomaterials showed higher water permeation flux and higher separation efficiency related to the pure PPSU membranes, while the rejection of PhP remained nearly constant of 94%. The pure water flux of rGO/TiO_2_-PPSU membranes was 348.6 L/m^2^h, being 119% higher than that of PPSU membranes (159.3 L/m^2^h), 17% higher than that of GO-PPSU membranes (298.5 L/m^2^h) and 10% higher than that of TiO_2_-PPSU membranes (315.9 L/m^2^h). The results mainly caused by the following two parameters: 1) The introduction of the membrane matrix with GO, TiO_2_ and GO/TiO_2_ nanomaterials with hydrophilic groups would endow the membrane excellent hydrophilicity, which was conducive to transport the water molecules across membranes (Abdel-Karim et al., 2021). As the results of contact angle shown in Figure 10B, the trend of improvement in contact angle was virtually consistent with the improvement of water permeability. 2) The appearance of porous structure of all the hybrid membranes resulted from the quick exchange between non-solvent and solvent during the phase transformation process was also beneficial to the water permeability promotion (Figure 2).

### Anti-fouling Performance and Reusability of the rGO/TiO_2_-PPSU Composite UF Membranes

**FIGURE 11:**
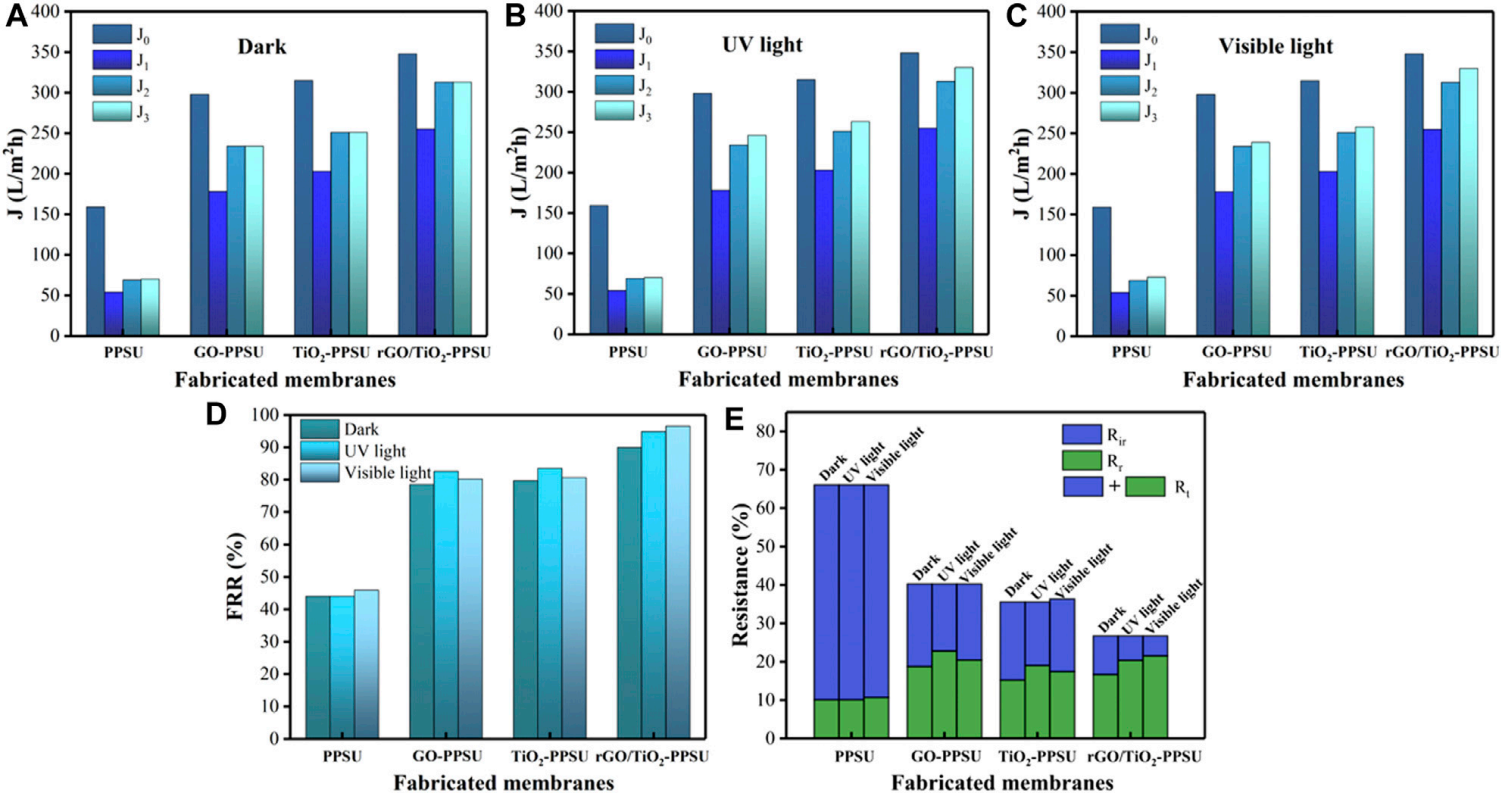
Equilibrium flux values at different steps (pure water, after fouling, water cleaning, and **(A)** darkness, **(B)** UV and **(C)** visible light irradiation) for membranes; **(D)** flux recovery ratio (FRR), **(E)** the total fouling ratio (R_t_), reversible fouling (R_r_) and irreversible fouling (R_ir_) of membranes in darkness, as well as under UV light and sunlight.

**FIGURE 12:**
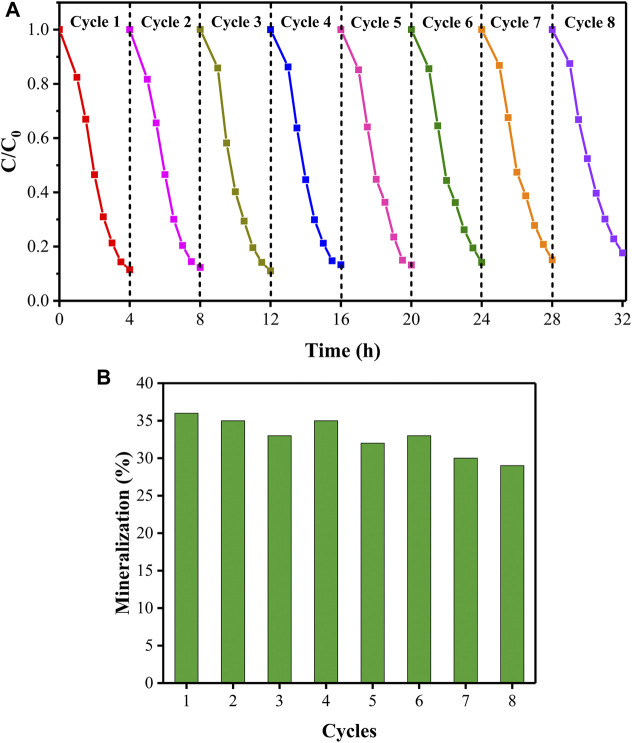
The removal rate **(A)** and mineralization rate **(B)** of PhP in the process of recycling under visible light irradiation (initial PhP concentration of 15 mg/L, initial pH of 8).

The antifouling and self-cleaning properties of all membranes were investigated using four-step filtration operations including before and after fouling by 15.0 mg/L PhP solution, after the cleaning by rinsing and the exposure in darkness, as well as under UV light and visible light, as shown in Figure 11. Obviously, the PhP flux presented significant decline due to fouling compared with pure water flux. However, the downward trend of the flux of the hybrid membrane was slower than that of the pure membrane, which can be attributed to the repulsive force between the pollutant and the membrane surface due to the increased electronegativity, indicating the enhancement of antifouling performance. The membrane permeate flux showed a limited recovery after removing loosely bound PhP with a backwash by straightforward distilled water. In order to further remove the fouling firmly adhering to the membrane surface and obtain a greater degree of flux recovery, the membranes were revealed to UV and visible irradiation, while the membrane was placed under dark conditions as a control group. Figure 11A showed the flux of all membranes have barely increase significantly after treatments. Nevertheless, after UV irradiation, the flux of all the hybrid membranes played further increased (Figure 11B), while after visible light irradiation, the flux recovery of the GO-PPSU and TiO_2_-PPSU membrane was limited (Figure 11C). Compared with GO-PPSU and TiO_2_-PPSU membrane, as showed in Figure 11D, the rGO/TiO_2_-PPSU membrane exhibited the superior flux recovery rate under UV and visible irradiation, which was similar to the photocatalytic degradation trend of PhP observed previously (Figure 8). The higher FRR, the better anti-fouling ability. In Figure 12E, the R_ir_ of PPSU membrane was 55.97, 55.89 and 55.35%, respectively; the R_t_ was 66.03, 65.95 and 66.04%, respectively. High values of R_ir_ and R_t_ mean more resistant fouling on the PPSU membrane surface, which cannot be removed after water washing and light irradiation. However, there was a sharp decline in R_ir_ and R_t_ with GO-PPSU, TiO_2_-PPSU and rGO/TiO_2_-PPSU membranes after UV and visible light irradiation. Among them, the R_ir_ and R_t_ of rGO/TiO_2_-PPSU membranes were minimum, R_ir_ values 10.06, 6.32 and 5.17%, respectively; R_t_ values 26.76, 26.75 and 26.72%, respectively. The phenomenon suggested that photocatalytic activity and the photo-induced hydrophilicity of GO/TiO_2_-PPSU membranes under UV and visible light could facilitate the removal of strongly bound PhP and endow the membrane with self-cleaning performance.

**FIGURE 13:**
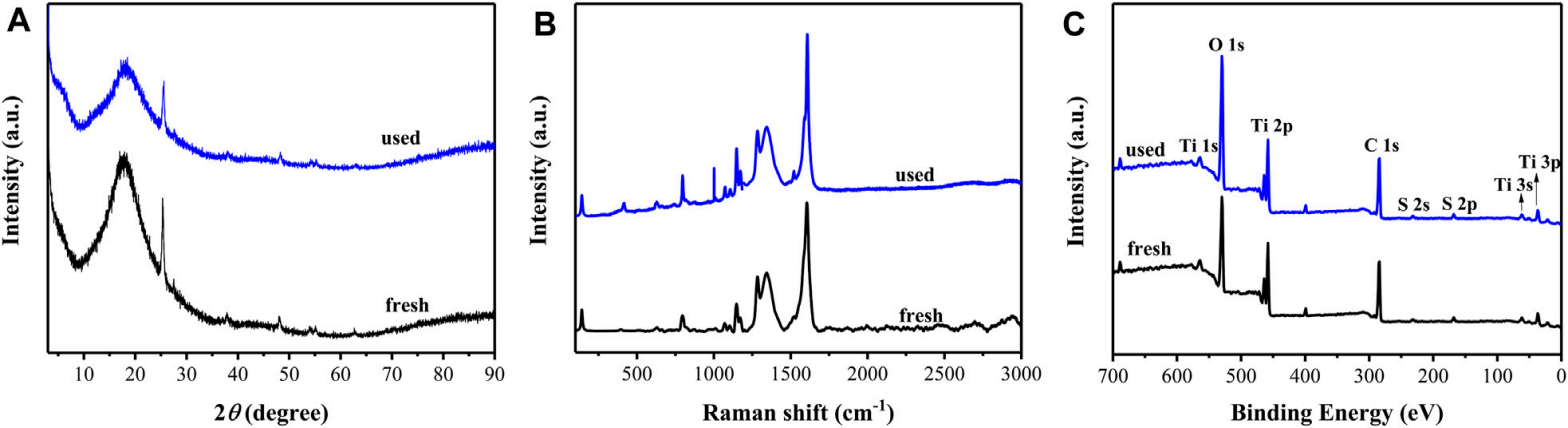
XRD **(A)**, Raman **(B)** and XPS **(C)** patterns of rGO/TiO_2_-PPSU hybrid membrane used before and after.

The reusability of the rGO/TiO_2_-PPSU hybrid membranes was examined at the same experimental conditions. Figure 12 illustrated that the photodegradation activity of the membranes maintain at 80% degradation rate (decreased about 7%) and the mineralization rate of PhP tended to decreased slightly (5.9%) after operation of eight cycles. To explore the anti-poisoning properties of rGO/TiO_2_-PPSU membranes, we carried out XRD, Raman and XPS measurements to observe the phase structure changes before and after operation of eight cycles. There were no obvious variations in XRD (Figure 13A) and Raman patterns (Figure 13B). In XPS spectra (Figure 13C), the intensity of C1s and O1s peaks of used rGO/TiO_2_-PPSU membrane was found to be slightly stronger than that of fresh one due to the adsorption of PhP degradation intermediates on it. Taken together, the rGO/TiO_2_-PPSU membrane performed well reusability and anti-poisoning properties.

## Conclusion

In this work, a novel rGO/TiO_2_-PPSU hybrid ultrafiltration membrane with enhanced self-cleaning and photodegradation property was fabricated via a non-solvent induced phase-separation method by adding rGO/TiO_2_ nanocomposites. The rGO/TiO_2_-PPSU composite membrane acquired a higher PhP degradation efficiency than pristine PVDF, GO-PPSU and TiO_2_-PPSU membrane under both UV and visible light. Moreover, the rGO/TiO_2_-PPSU hybrid ultrafiltration membrane was endowed with a higher permeation flux (348 L/m^2^h) and water flux recovery rate (>90%) under simulated visible light irradiation due to better self-cleaning performance of the membrane. Moreover, the composite membrane demonstrated an outstanding reusability with a slight drop of photocatalytic efficiency after eight cycles.

## Data Availability

The original contributions presented in the study are included in the article/Supplementary Material; further inquiries can be directed to the corresponding authors.
